# Microbial Contamination of Dental Unit Waterlines and Potential Risk of Infection: A Narrative Review

**DOI:** 10.3390/pathogens9080651

**Published:** 2020-08-13

**Authors:** Anna Maria Spagnolo, Marina Sartini, Maria Luisa Cristina

**Affiliations:** Department of Health Sciences, University of Genova, 16132 Genova, Italy; am.spagnolo@unige.it (A.M.S.); cristinaml@unige.it (M.L.C.)

**Keywords:** dental infection control, water microbiology, biofilms

## Abstract

Several studies have revealed that dental unit waterlines (DUWLs) are often contaminated by large numbers of various micro-organisms (bacteria, fungi, protozoa, viruses). Microbial contamination in DUWLs may originate from the mains water piped into the dental unit, the suck-back of patients’ saliva into the line due to the lack of adequate valves, and contamination from bottled water systems. Some of the main determinants of microbial contamination in DUWLs are: a very small lumen size (0.5–2 mm) of the tubing used, high surface-to-volume ratio (6:1), low throughput and the materials of which the tubing is made, water stagnation outside of working hours. The environmental conditions present inside the conduits of the dental unit may facilitate the proliferation of micro-organisms and the consequent formation of biofilm on the interior surface of the pipes of DUWLs. During the use of handpieces, particularly high-speed rotating instruments, a spray is thrown up in the form of aerosols or spatters containing biological material (saliva, blood and dental plaque) and micro-organisms. This means that the health of both dental staff and patients could be at risk of infection. The risk of cross-infections in dental settings can be tackled by implementing combined interventions to prevent the contamination of DUWLs.

## 1. Introduction

### 1.1. Background

**Figure 1:**
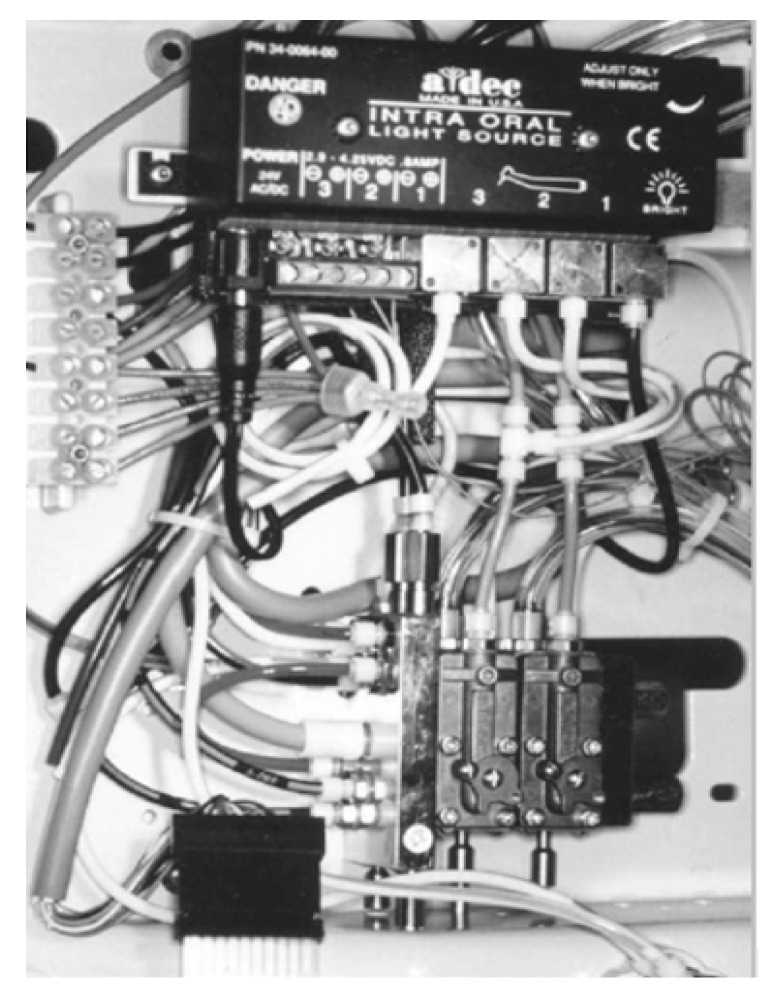
Part of the interior of a dental unit (reproduced with permission from Marshall GW Jr [5]).

Dental treatment requires various essential services, such as electric power, suction, air and water supply, which are provided by the dental unit. During treatment, high-speed rotating instruments and ultrasonic scalers generate heat, which can damage dental tissues. To avoid overheating, these instruments are cooled by water, which may be supplied by the municipal water-main or by tanks (bottles) directly connected to the dental unit. This water, which is also used to irrigate the operating field, travels to the instruments of the dental unit through a complex network of narrow-bore plastic tubes, valves and connectors: the so-called dental unit waterlines (DUWLs) [1]. This network conveys water to the high-speed handpiece, air/water syringe, and ultrasonic scaler (Figure 1), in order to cleanse, cool and rinse both the site of intervention and the equipment needed for work on soft and hard tissues [2,3].

Several studies have revealed that DUWLs are often contaminated by large numbers of various micro-organisms (bacteria, fungi, protozoa, viruses) [4,5,6,7]. Indeed, copious evidence of dental unit water contamination has accumulated since the 1960s, when water from DUWLs was first found to contain micro-organisms in concentrations ranging from 10^4^ to 10^6^ colony-forming units (CFU)/mL [8].

The Guidelines on infection control in dental healthcare settings issued by the US Centers for Disease Control and Prevention (CDC) recommend that the level of heterotrophic plate counts (HPCs) in dental unit water should not exceed 500 CFU/mL [9]. Moreover, the American Dental Association (ADA) has set a limit of ≤200 CFU/mL on the heterotrophic bacterial load in water from dental unit waterlines [10]. In the EU, however, there is no current guideline regarding DUWLs, though in some countries the drinking water standard is used as a reference [11].

Microbial contamination in DUWLs may originate from the mains water piped into the dental unit, the suck-back of patients’ saliva into the line due to the lack of adequate valves, and contamination from bottled water systems [12,13]. Thus, dental waterlines may be contaminated not only by water-borne environmental microorganisms, but also by germs from the oral cavity of the patient, which is host to microbes that belong to different species, such as *Porphyromonas gingivalis*, one of the bacteria implicated in the biofilm formation of bacterial plaque [14,15]. 

Indeed, dental turbines rotate at high speed and, during the slowdown phase, exert an aspirating effect, which causes organic material present on the tip of the instrument to be drawn inside, thereby giving rise to the phenomenon of back-contamination of the water supply [16]. This contaminated water may contain bacterial loads that are thousands of times greater than the limits set on drinking water. Thus, even the most scrupulous sterilization of the handpieces of the dental unit will be in vain if these instruments are connected to a contaminated water supply.

Some of the main determinants of microbial contamination in DUWLs are: a very small lumen size (0.5–2 mm) of the tubing used, high surface-to-volume ratio (6:1), low throughput and the materials of which the tubing is made. Indeed, during working hours, the water flows, albeit for brief periods; outside of working hours, however, the water stagnates. Together, these factors offer the micro-organisms present in the water ample opportunities to build up a strong matrix-encapsulated biofilm that can resist antimicrobial treatments [1,11,12,17,18,19]. This means that the health of both dental staff and patients could be at risk if water is not appropriately treated [20].

### 1.2. Aim

The aim of this narrative review is to describe microbial contamination of dental unit waterlines and the potential risk of infection. Searches were made in PubMed.gov and Scopus for publications regarding dental unit waterlines, DUWLs, related to microbial contamination, biofilm and infection risk prevention.

## 2. Microbial Contamination of DUWLs and Biofilms

Many micro-organisms (bacteria, viruses, fungi) have been found in water samples from DUWLs: *Streptococcus mitis, Streptococcus salivarius, enterococci, Staphylococcus cohnii, Staphylococcus warneri A, Klebsiella (Enterobacter) aerogenes, Bacillus subtilis, Enterococcus faecalis, Enterobacter cloacae, Pseudomonas aeruginosa, Legionella pneumophila, Serratia marcescens, Aeromonas* spp., *Acinetobacter* spp., *Flavobacterium* spp., *Moraxella* spp., *Cladosporium* spp., *Pseudomonas* spp., *Legionella* spp., etc. [4,5,6,7].

Abdouchakou et al. [21] reported the pattern of microbial contamination of waterlines over 6.5 years in a dental healthcare center in which 61 dental unit waterlines (DUWLs) were connected to the same water supply. One clone of *P. aeruginosa* and 2 clones of *Achromobacter* sp. gradually colonized all the DUWLs; the last colonization by *P. aeruginosa* ST309 prompted the closure of the dental care center. Moreover, confirmation of the literature reports was provided by a recent study [22] which found *Legionella* and *P. aeruginosa* contamination in 32% (6/19) and 68% (13/19), respectively, of water samples from DUWLs. In addition, the presence of Gram-negative bacteria in DUWLs can lead to the production of endotoxins (LPS) in the water and air of a dental surgery [23].

Fungi have also been found in water from dental units. In a study conducted by Mazari et al. [24], 18 dental waterlines were analyzed for the presence of yeasts on their internal surfaces. Of the 18 DUWLs studied, 10 were contaminated (55.56%). *Candida albicans*, *Candida guilliermondii* and *Candida glabrata* and two species of non-Candida, *Rhodotorula* spp. and *Trichosporon* spp., were identified.

In addition to bacteria, fungi and viruses, protozoa such as free-living amoebae have been isolated from DUWLs [25,26]. These can act as a reservoir for micro-organisms (e.g., *Legionella* spp. *Pseudomonas* spp. etc.) or as pathogens in their own right [27,28].

In our previous study [29], we assessed the level of contamination by bacteria and amoebae in 30 dental units, all of which were supplied directly by the municipal water network and no additional disinfection systems were used in the facilities. The mean concentration of HPCs at 22 and 36 °C was 1168.53 CFU/mL and 827.90 CFU/mL respectively, while the concentration of *P. aeruginosa* proved to be of 25.13 CFU/100 mL. Of the 30 units, 26.67% displayed a concentration of ≥10^3^ CFU/L of *L. pneumophila*; about 23% of cases involved *L. pneumophila* sg 1. The study revealed that the water in the DUWLs contained considerably higher concentrations of micro-organisms than the input water supply, thereby confirming the role of the water system inside the dental unit in increasing microbial contamination, especially in the absence of proper management of the water and healthcare risk.

Previous studies have found a wide range in the rate of recovery of Legionella contamination of DUWLs, from 0% to 100% of DUWL systems [30,31] including *Legionella pneumophila* serogroup 1 [32], reaching levels as high as 10^5^ colony-forming units per milliliter [30,33,34]. The presence and concentration of *Legionella* contamination in DUWLs varies according to the features of the water supply system, the design and model of the dental unit, and the methods of disinfection used [30].

## 3. Formation of Biofilms in DUWLs

**Figure 2:**
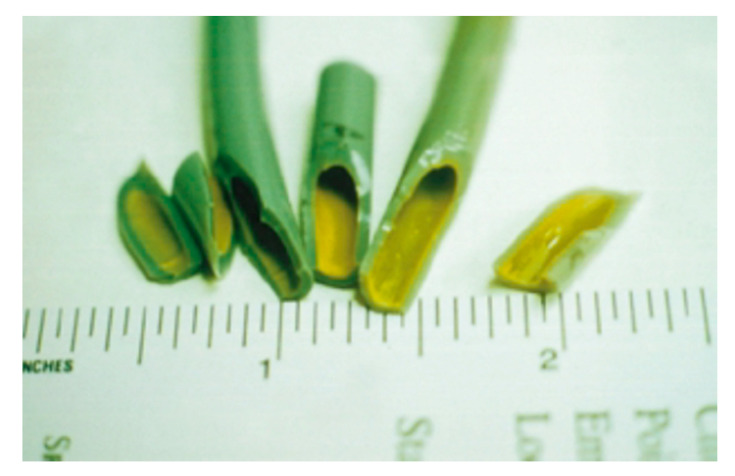
Biofilm formation on dental unit waterline tubing (reproduced with permission from Mills SE [41]).

**Figure 3:**
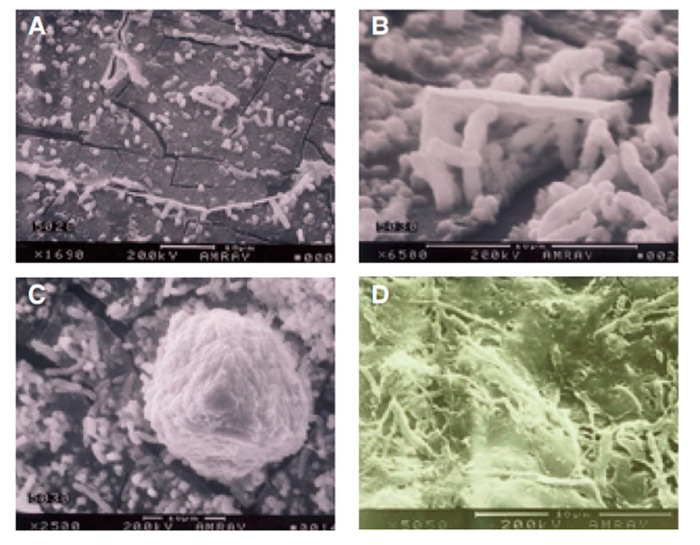
Phases of biofilm formation on the surface of a dental unit waterline (reproduced with permission from Mills SE [41]): (**A**) carbonate deposits (which resemble ice floes); (**B**) initial attachment; (**C**) division of cells into microcolonies; (**D**) formation of biofilm.

The proliferation of micro-organisms and the consequent formation of biofilm on the interior surface of the pipes of DUWLs (Figure 2 and Figure 3) may be facilitated by the environmental conditions present inside the conduits of the dental unit.

The formation of biofilm in DUWLs is a universal problem, as is indicated by the results of many studies in several countries. Biofilms constitute the main reservoir for continued contamination of water supply systems [35]. They are made up of bacteria that adhere to the surface of a substrate and which are embedded in a matrix of extracellular polymeric substances (EPS) [36]. As EPS may constitute 50–90% of the total carbon present in biofilms, they can therefore be regarded as the primary matrix. Although their chemical and physical properties vary, EPS are chiefly composed of polysaccharides and proteins, which form highly hydrated matrices. The micro-organisms in the biofilm can maintain a stable synergy among different species, thereby orchestrating the degradation of complex substrates. Inside the biofilm, micro-environments are created which are characterized by physical and chemical gradients in which a fundamental role is played by the EPS. As the matrix can sequester nutrients from the environment, it plays a part in the general strategy for survival in oligotrophic conditions. The micro-organisms contained in the biofilm display resistance to biocidal agents that is 2–3-orders of magnitude greater than that of non-adherent microbial cells [37].

Thus, biofilms form as a result of a process of microbial attachment to the substrate, cell proliferation and matrix production, which may be followed by detachment [38,39,40,41] (Figure 3). Indeed, during the normal use of the dental unit, portions of biofilm may detach from the substrate and be conveyed to the external environment through the sprays of handpieces.

Tall et al. [42] conducted a 6-month study of bacterial colonization and biofilm formation in plastic waterlines connected to dental air/water syringes. During the period of observation, changes in biofilm flora were observed by means of both scanning electron microscopy and bacteriological culture. After 6 months, several layers of morphologically different micro-organisms were detected, which completely covered the lumen. The authors presented the succession of species in order of appearance, as cultured. Early colonizers were mainly *Pseudomonas* spp.; later, *Pasteurella*, *Moraxella*, *Ochrohactrum*, *Aeromonas* spp., *Flavobacterium* and *Acinetobacter* spp. were observed.

## 4. Risk of Infection from DUWLs

The risk of infection resulting from dental treatment is a public health concern, especially since such treatment is extremely common in the population and increasing numbers of medically compromised or immunocompromised individuals (for various reasons: chronic diseases, AIDS, cystic fibrosis, immunosuppressive therapy for organ transplantation and chemotherapy, etc.) are receiving regular dental treatment.

Fortunately, few published studies concern cases of infection resulting from dental care. Nevertheless, there is a risk that large numbers of pathogenic micro-organisms may be swallowed, inhaled or inoculated into oral wounds during dental treatment, potentially giving rise to colonization or infection [43].

In dental settings, infections may spread directly through contact with blood, oral fluids or other secretions; indirectly, through contact with contaminated instruments, equipment or environmental surfaces; and through inhalation or contact with micro-organisms present in aerosols or spatters of oral and respiratory fluids or in dental unit waterlines [23,44]. Moreover, back-pressure phenomena created by rotating instruments play a major role in the retrograde aspiration of contaminated fluids from the oral cavity into DUWLs. These fluids may then be sprayed onto the mucosae of the next patient treated, even if the handpiece has been correctly replaced with a sterilized one and sterile water is used for irrigation [45]. Thus, during the use of handpieces, particularly high-speed rotating instruments, a spray is thrown up in the form of aerosols or spatters containing biological material (saliva, blood and dental plaque) and micro-organisms [46,47]. This cloud, which comes directly into contact with dental staff and patients, is made up of material that partly originates from the treatment site and partly comes from dental unit waterlines. The aerosol produced can remain airborne for long periods and may be inhaled by dental staff and patients; it then tends to sediment, contaminating surfaces, operating equipment, etc., thereby increasing the risk of infection [44,48].

Anyway, some methods can be used to reduce aerosols contaminating load, such as mouth-rinses with chlorhexidine, cetylpyridinium chloride, hydrogen peroxide and other bactericide and virucide agents, the use of the dental dam and of the high-volume evacuator.

The aerosols thrown up by the rotating instruments used in dentistry were analyzed in a study carried out by Miller; this revealed that particles ranging from 0.06 to 13 µm in diameter, and with a half-life of 35 min to 17 h, were produced [47,49].

Rautemaa et al. [50] determined both how far airborne bacteria travelled during dental treatment and the level of contamination at various distances. To this end, they collected fall-out samples on blood agar plates situated at six points at distances of 0.5–2 m from the patient. Significant contamination was recorded at all sampling points when high-speed instruments were used (mean concentration on surfaces per hour: 1119 CFU/m^2^/h at >1.5 m from the patient).

Instead, Zemouri et al. [51] in a more recent study, have found a contamination up to 655 CFU/plate/30 min.

Prospero et al. [52] evaluated the sedimenting bacterial load in dental surgeries during routine procedures. The surfaces with the highest levels of contamination were, in descending order, dental healthcare workers’ surgical masks, lamps, areas near spittoons, and mobile trays. Positive samples taken from the various surfaces presented *Streptococcus* species (42%), *Staphylococcus* species (41%) and Gram-negative bacteria (17%).

With regard to Gram-negative bacteria, *Pseudomonas aeruginosa* is frequently studied in waterlines because of its association with disease in susceptible hosts (e.g., subjects with cystic fibrosis). These pathogens are easily transmitted and originate from the main water pipes. A study conducted by Jensen et al. [53] assessed the presence of *P. aeruginosa* in samples of water taken from triple-function syringes, turbines, handpiece contra-angles and ultrasonic scalers during dental treatment sessions attended by cystic fibrosis patients. In addition, samples of expectorate taken from each patient affected by cystic fibrosis (CF) were examined for the presence of *P. aeruginosa* each month, before and after each treatment session. At least in one case, genotypically identical (RFLP, pulsed-field gel electrophoresis) *P. aeruginosa* strains were found both in water from the dental equipment and in the CF patient’s sputum. In such patients, exposure to *P. aeruginosa* must be avoided, as this micro-organism is the primary cause of lung destruction and premature death in these patients [54].

Other Gram-negative bacteria frequently encountered in DUWLs are Legionellae. This micro-organism grows at temperatures of 25–50 °C, especially in water that is stagnant and rich in sediments, and causes various clinical manifestations, including the form of pneumonia commonly known as Legionnaires’ disease [55,56,57].

Fortunately, there are few documented cases of legionellosis acquired through a dental unit. In one such case, however, an 82-year-old Italian woman died of pneumonia after exposure to *Legionella pneumophila* serogroup 1, which was detected in the DUWL. Molecular typing confirmed the clonal relationship between the clinical and environmental strains [58]. Another case of legionellosis linked to dental treatment was reported in an immunocompromised elderly Swedish man who developed Legionnaires’ disease a few days after a dental check-up. *Legionella* spp. at a concentration of 2000 CFU/L was isolated from the outlet of the water used for oral rinsing. Isolates from the patient’s sputum, collected by means of bronchoscopy, and from the dental unit were of *Legionella pneumophila* sg 1, subgroup Knoxville and ST9 [59]. Other species, such as *Achromobacter* species and mycobacteria, have also been associated with infections from waterlines [23,60].

Exposure to bacteria from DUWLs affects not only patients but also dental staff. The occupational risk of Legionella infection is not a marginal issue, since a high number of individuals could be occupationally exposed [61]. In this regard, higher rates of seropositivity for Legionella antibodies have been found among dental personnel than among the general public [62,63], suggesting that the aerosols generated in dental surgeries are a source of exposure to *Legionella* spp. Moreover, those who spend more years in dental practice have a higher risk [30].

In 1985, a study performed in a US dental school [63] reported the prevalence of Legionella-specific IgG and IgM antibodies in a dental clinic population consisting of 270 dental school personnel. Subjects were subdivided into two groups: those exposed for over two years (Group One) and those exposed for one year or less (Group Two). A control group of 67 individuals without previous clinic exposure was randomly selected from the regional population (Group Three). The incidence of IgG-positivity was highest in Group One (23%), lower in Group Two (16%), and lowest (8%) in Group Three (no exposure to the clinic environment).

Regarding cases of professionally contracted legionellosis, the literature documents only one fatal case of Legionnaires’ disease in an American dentist. This infection was attributed to exposure to DUWL aerosols. *L. pneumophilia* and *L. longbeachae* were detected in the dentist’s lung tissue and in the DUWLs; however, the dentist’s domestic water supply also presented *Legionella* spp., albeit at very low levels [22,64].

Given that the literature data indicate ample contamination of dental unit waterlines by Legionella, it is recommended that sampling for the detection of this micro-organism be carried out every time that a case of disease occurs, and at least once a year if risk-minimization measures are not implemented [65].

## 5. Treatment of DUWLs

The risk of cross-infections in dental settings can be tackled by implementing appropriate systems of proven efficacy. There are different chemical, physical or chemo-physical treatment systems of DUWLs, (e.g., peracetic acid, gluteraldehyde, chlorhexidine, chlorine dioxide, filtration, flushing, reverse osmosis, etc.), [2,16].

According to the guidelines of Italian Health Ministry for the prevention and control of legionellosis [65], in order to reduce microbial contamination and/or the formation of biofilm in DUWLs, the following recommendations should be implemented:

1. Any sections excluded from the flow currents should be eliminated from the network.
2. Install anti-stagnation devices to keep the water circulating continuously, particularly during non-working hours.
3. Supply the network with sterile solutions, after isolating it from the main water supply.
4. Disinfect the water by means of continuous or discontinuous treatments. These latter, which may be carried out periodically or between one patient and the next, prevent chemical contamination of the operating field, reduce the exposure of staff and minimize the risk of selecting resistant micro-organisms; however, they require a greater commitment of resources and attention than continuous treatments.
5. All devices that connect to a waterline and enter patients’ mouths, such as handpieces, ultrasonic scalers and air/water syringes, should be switched on and flushed through before use: for at least two minutes at the beginning of each working day and for at least 20–30 s before each patient is treated.
6. Filters (≤0.2 μm) that can trap micro-organisms coming from inside the water supply network should be installed immediately upstream of handpieces.

In addition, in the case of invasive surgical procedures, only sterile water should be used, and the supply network should also be sterile. If sterility of the dental unit’s supply network cannot be guaranteed, a bypass system should be created and disposable sterile devices, or sterilizable devices, should be used [9,65].

Various international agencies also recommend implementation of these preventive measures, the efficacy of which has been demonstrated by several scientific studies [6,45,66,67].

The Centers for Disease Control and Prevention (CDC) [9] recommend that manufacturers should provide dental units with a separate reservoir, typically a container of about 1-liter capacity, from which tap water, deionized water and/or distilled water can be fed to the drill in order to cool it. Moreover, these separate reservoirs are also better suited for the input of biocides. This measure was introduced by the CDC in dentistry in order to supply safe treatment water, although some working dental units are still fed directly by municipal water [7]. In these latter cases, it is even more important to adopt the various systems for preventing microbial contamination, such as, for example, the use of handpieces and turbines fitted with anti-reflux valves; these valves are triggered when the turbine stops and prevent liquids, and hence also micro-organisms, from being aspirated when rotating instruments are used.

Particularly recommended by the CDC [9] is flushing, which should always be carried out for 20–30 s after each patient is treated. This should be done for all devices that connect to a waterline and enter patients’ mouths, such as handpieces, ultrasonic scalers and air/water syringes. This procedure is intended to physically flush outpatient material that might have entered the turbine, air or waterlines. A recent study [6] evaluated the impact of flushing on bacterial contamination in dental unit waterlines. Water samples were collected (before flushing, 1 min post-flushing, and 3 min post-flushing) from 24 clinics (Group A: no disinfection, Group B: citric acid disinfectant). High-speed drill handpiece (HP) lines, air/water syringes (WS) and oral rinse (OR) were monitored. All sources tested (HP, OR and WS) from Group A units showed significant levels of bacterial contamination, while no contamination was observed in the water samples from Group B clinics. The bacterial contamination of WS and HP samples was significantly lower 3 min after flushing than before flushing, but was still higher than the recommended limit of 500 CFU/mL. This suggests that flushing alone is not sufficient and that other strategies are required in order to improve water quality in dental practices.

DUWL biofilms may be treated with biocides either periodically or by means of continuous treatment systems. Continuous disinfection systems are more effective and easier to comply with, and require less organization. The chemical agents currently used for disinfecting DUWLs include chlorine dioxide, sodium hypochlorite, hydrogen peroxide and chlorhexidine gluconate. Optimal disinfectant products should be economical and have few side-effects on dental units and the oral cavity. Disinfectants containing sodium hypochlorite or hydrogen peroxide, which have shown the greatest efficacy, are, however, reported to have side-effects, such as corrosion of dental equipment, clogging of the DUWLs, and stimulation of the oral mucosa [68]. Finally, because disinfectants are tested in vitro for registration and the construction features of DUWLs vary widely, field tests and disinfection protocols are tailored to individual devices. It is therefore unusual to find a disinfection protocol that is effective in all cases [31].

In conclusion, reducing the risk of infection in dental-care settings depends on the implementation of combined interventions to prevent the contamination of DUWLs. Equally important, however, are the scheduled technical maintenance of dental units and the monitoring of the quality of DUWL water. Moreover, recommended transmission-based precautions should be incorporated into daily practice, in order to minimize aerosol production through the use of high-velocity air evacuation and air conditioning systems.
